# A new family of modified Gaussian copulas for market consistent valuation of government guarantees

**DOI:** 10.1007/s11846-022-00600-1

**Published:** 2022-10-25

**Authors:** Roy Cerqueti, Francesco Cesarone, Maria C. Heusch, Carlo D. Mottura

**Affiliations:** 1 Department of Social and Economic Sciences, Sapienza University of Rome, Piazzale Aldo Moro, 5, 00185 Rome, Italy; 2 School of Business, London South Bank University, 103 Borough Rd, London, SE1 0AA UK; 3 GRANEM, Universitè d’Angers, 49036 Angers CEDEX 01, France; 4 Department of Business Studies, Roma Tre University, Via Silvio D’Amico, 77, 00145 Rome, Italy

**Keywords:** Gaussian copulas, Stochastic dependence, Default risks, Government guarantee, Financial crisis, 62H05, 91-10, 91G30

## Abstract

This paper deals with a copula-based stochastic dependence problem in the context of financial risks. We discuss the financial framework for assessing the theoretical up-front value of government guarantees on bank liabilities. EU States widely use these contracts to improve the financial system’s stability and manage the banking sector in crisis situations; in Italy, they have also been used to address the consequences of the Covid-19 emergency. From a market viewpoint, we deal with a defaultable guarantee contract where the State-guarantor and the bank-borrower are both subject to default risk, and their risks are interconnected. We show that the classical Gaussian copula is not satisfactory for modeling the dependence among the considered risks. Indeed, using the benchmark market model for credit risk portfolio management, we highlight some contradictory results observed for the up-front values of the guarantee when the default intensity of the guarantor is smaller than that of the borrower. Then, we introduce a new family of modified Gaussian copulas that overcomes the limitations of the standard approach, allowing to determine realistic results in terms of the guarantees “mark-to-model” value when the benchmark market model does not work. Numerical simulations validate the theoretical proposal.

## Introduction

After the global financial crisis that began in 2008, States has widely used government guarantees to improve the stability of the financial system and to restore market confidence in a systemic crisis situation (Allen et al. 2018; LaBrosse et al. 2013; Levy and Zaghini 2010; Communication from the Commission 2008, 2011, 2013).

In Italy, these guarantees have also been used to address the consequences due to the Covid-19 emergency (see, Italian government 2020a, b). In the EU framework, the assessment of the theoretical up-front value of government guarantees on bank liabilities are a relevant issue: government guarantees can be used as “stability” financial instruments only if their values are “market consistent”; otherwise, given the prohibition of State aids, they are not allowed (see Sect. 2 in Bassan and Mottura 2015).

In particular, the EU Commission indicates the “ex-lege” methodology for calculating the guarantee fee on a backwards-looking basis; but no indication is given for an ex-ante fee valuation (i.e., on a forward-looking basis).

We examine here the problem of evaluating a guarantee contract where both the guarantor and the borrower are subject to default risk, and these risks are correlated. In particular, we tackle the problem of financial valuation of *government guarantees on bank liabilities* in the broader context of the institutional measures used by the EU States to improve the financial stability of markets and restore confidence in the banking sector. The EU Commission has indicated the methodology for calculating fees for banks that benefit from such guarantees, requiring that they should be “market consistent” (Communication from the Commission 2008).

The issue of stochastic dependence arises from the connections between the banks and the State. On the one hand, the bank plays the role of underwriter of the securities issued by the State, so that such securities are assets on banks’ balance sheet. If the default risk of the State increases, then the value of banks’ investment reduces and the default risk of banks increases. On the other hand, any bank’s failure generates an increase of the risk of refinancing the government debt, thus the default risk of the State. This is the problem of dependence or circularity of default risk between State and bank.

The identification of the nature of the correlation among the involved risks represents a challenging aspect of the problem and offers room for theoretical additions to the related literature. We here deal with such a methodological theme by introducing a suitably defined new family of copulas (for a survey on copulas, see Joe 2014; Nelsen 2007).

Indeed, the classical Sklar’s Theorem (see Sklar 1959) assures that copulas can capture the stochastic dependence among a set of random quantities. In this respect, the large number of existing copulas provides rather complete coverage of the possible types of association, ranging from tail dependence (see, e.g., Dolati et al. 2014; Fernandez-Sanchez and Ubeda-Flores 2019) to generalized concepts of positive dependence, like the multivariate total positivity of order 2 (see, e.g., Cerqueti and Lupi 2016; Wysocki 2015) and the positive quadrant dependence (see, e.g., Gijbels et al. 2010; Saminger-Platz et al. 2021).

In our context, for financial markets this *defaultable guarantee* can be interpreted as *defaultable single name Credit Default Swap* (CDS, see Mottura and Passalacqua 2013a, b, 2014; Liang et al. 2014). It follows that in our financial valuation analysis the reference defaultable guarantee has been valued under the standard Gaussian copula model, which is the benchmark for such credit derivative (see, e.g., Brigo and Chourdakis 2019; Chen et al. 2012; Morini 2011). One of the main reasons for the widespread use in the industry of the Gaussian copula is the easy interpretation of its linear correlation parameter. Moreover, the Gaussian copulas represent one of the main relevant examples of association measures, being suitable for describing either positive as well as negative dependence (see, e.g., Masarotto and Varin 2012; Pitt et al. 2006). In this respect, it is important to notice that proper selections of the correlation parameter let the Gaussian copula attain the Frechet upper and lower bounds and the independence case of the product copula in the bivariate case (see Joe 2014; Xue-Kun Song 2000).

This explains the popularity of such methodological tools and their extensions in financial applications (see, e.g., Malevergne and Sornette 2003).

Nevertheless, the original version of the Gaussian copula exhibits crucial limitations; hence they are sometimes inappropriate for modelling some special types of financial dependence structures. Zimmer (2012) elaborates on such inconsistency by presenting the paradigmatic case of the housing crisis as an extreme event. In Fang and Madsen (2013), the authors offer a modified version of the Gaussian copula for financial and insurance contexts. In the same line, Furman et al. (2016) encourage *“substitution of the Gaussian copula with other copulas”*.

This paper contributes to this scientific debate. We take for us the attitude of the Gaussian copulas to describe the dependence among financial risks, but we overcome their limitations by presenting a novel family of copulas that is a modification of the standard Gaussian ones.

To provide a model that allows determining “realistic” results in terms of the guarantees “mark-to-model” value when the Gaussian model does not work, we suggest a model that gives rise to values of the guarantee with the following three *conditions of consistency*: (1) the up-front values of the guarantee must always decrease with the increase of the dependence parameter between the default times of the parties; (2) for any fixed level of the dependence parameter, the values of the guarantee have to be decreasing with the intensity of default of the guarantor ($\lambda _1$), given the intensity of default of the borrower ($\lambda _2$), and have to be increasing with $\lambda _2$, given $\lambda _1$; (3) the up-front value of the guarantee has to be equal to 0 in the case of a perfect positive correlation between the default times of the parties.

From a methodological perspective we follow a “best effort” principle, which ensures the best compromise between the mathematical tractability and the meaningfulness of the guarantee’s values. Specifically, we provide an exogenous adjustment of the Gaussian copula (that we call *Modified Gaussian* (MG) copula/model) by combining the *standard* Gaussian copula with the default time distributions of the parties where the default intensities of the guarantor are appropriately modified by an “adjustment function”. This adjustment function is defined as a deterministic “S” shaped function of three parameters. Two of them can be set to produce values of the guarantee contract by the MG model as close as possible to those of the Gaussian model, when the level of the dependence parameter of the Gaussian copula is smaller than a prefixed maximum acceptable correlation; the other adjustment parameter is a strategic (exogenous) variable in the hand of the calculation agent. Clearly, when the “adjustment function” approaches zero, the MG model collapses on the Gaussian one.

We point out that the introduction of the adjustment function mitigates the symmetry derived by the employment of the Gaussian copula, in a context which is generally asymmetric. Indeed, the deviation between the behavior of the default probabilities of State and banks is a stylized fact of several markets.

Extensive simulations based on real data validate the theoretical proposal.

The structure of this paper is as follows. Section 2 introduces the considered financial setting, with some preliminary concepts on the reference guarantee contract. The notations used in this section are also those employed in the methodological part of the paper. Section 3 is devoted to the valuation of the reference guarantee by using the standard Gaussian copula model; such a section provides also some numerical results and a discussion of the main drawbacks of the standard Gaussian approach. In Sect. 4, we propose a new model (the MG model) to overcome the shortcomings of the Gaussian model. Finally, Sect. 5 contains some concluding remarks and traces lines of future research.

## General framework

In this section, we introduce the financial framework for assessing the theoretical up-front value of *government guarantees on bank liabilities*. This financial valuation will refer to a defaultable guarantee contract where the State-guarantor and the bank-borrower are both subject to default risk, and their risks are interconnected (*reference guarantee contract*).

For completeness, we recall some well-known financial concepts related to this point.

All the operators in financial markets are risky, i.e., exposed to “default risk”, which can be measured in terms of Credit Default Swaps (CDSs) prices.

The CDSs are derivative instruments in which a party, in return for payments to the counterparty, protects itself against the risk of default associated with a particular debtor (reference entity). The debtor may be a company or a State issuing a bond. CDSs are traded over-the-counter, and their prices indicate the cost of hedging the underlying debtor’s default risk per notional value. In this sense, CDSs are normally used as a “risk thermometer” to measure the level of debtors’ risks.

In the interbank credit derivatives market, a risky guarantee corresponds to a single-name defaultable CDS: the reference entity is the bank debt; the protection seller is the guarantor State; the protection buyer is the issuing bank, which pays the guarantee fee to the protection seller. In the event of default of the reference obligation, the repayment to the bondholders by the protection seller is the default payment leg of the CDS; the guarantee commission paid by the debtor is the premium payment leg of the CDS. Typically, interbank CDSs are collateralized; in this sense, they can be interpreted as market default-free guarantees.

When embedded in the “market,” the analyzed defaultable guarantee contract—between a single debtor and a single guarantor, in our case—is similar to a defaultable CDS, and relevant evaluation problems occur. Indeed, the European Central Bank has highlighted that when the creditworthiness of the reference entity of the CDS is correlated to the payment capacity of the counterparty of the CDS, a so-called wrong-way risk is produced, underlining the importance of controlling this type of risk. Quoting European Central Bank (2009): *“the increased correlation in the CDS market between reference entities and sellers of CDS protection lessens the effectiveness of clean transfer of risk and amplifies the effect of this interconnectedness. This risk, called wrong-way risk, occurs when a CDS reference entity’s creditworthiness or credit quality is correlated with the CDS counterpart’s ability or willingness to pay”*.

We can now go on with the mathematical description of the general framework.

The notation introduced in this section will also be used for describing the Gaussian copula approach, the drawbacks of such a standard setting and, finally, for introducing the novel concept of modified Gaussian copula (see the next sections).

We denote by $\tau _{1}\ge 0$ and $\tau _{2} \ge 0$ the times of default of the guarantor (party 1) and of the borrower (party 2), respectively, and we assume they are correlated. The guarantor pays the payoff of the guarantee $\Pi$ to a guaranteed party at the time of default of the borrower $\tau _{2}$ (if $\tau _{2}$ occurs before the maturity of the contract *T*), and is defined as follows

$$\begin{aligned} \Pi ({\tilde{\tau }}) = N[1-R({\tilde{\tau }})]{\mathbf {1}}_{\left\{ \tau _{1}>\tau _{2}\right\} }{\mathbb {\mathbf {1}}}{}_{\left\{ \tau _{2}\le T\right\} }, \end{aligned}$$

where *N* is the nominal amount of the contract, $R({\tilde{\tau }})$ is the stochastic recovery rate at time ${\tilde{\tau }}$, ${\tilde{\tau }}=\min \{\tau _{2},T\}$, and ${\mathbf {1}}$ is the indicator function. We assume a standard probability space $\left( \varOmega ,{\mathcal {G}},\{{\mathcal {G}}_{t}\}_{t \ge 0},{\mathbb {Q}} \right)$, where $\varOmega$ is the sample space, ${\mathcal {G}}$ is the $\sigma$-algebra, ${\mathcal {G}}_{t}$ is the relevant filtration representing the flow of information up to time *t*, and ${\mathbb {Q}}$ is the risk neutral measure. Under the no-arbitrage market condition, the theoretical up-front value of the guarantee *V*(*t*) at the valuation time *t* is given by

1 $$\begin{aligned} V(t)={\mathbb {E}}_{t}^{{\mathbb {Q}}} \left[ e^{-\intop _{t}^{{\tilde{\tau }}}r(u)du}\Pi ({\tilde{\tau }}) \mid {\mathcal {G}}_{t} \right], \end{aligned}$$

where *r*(*u*) is the risk-free interest rate intensity (spot rate) prevailing on the market at time *u*. We point out that to evaluate equation (1) we focus on the probability that the guarantee is paid $P\left[ (\tau _{2}\le T)\cap (\tau _{1}>\tau _{2})\right]$, namely, the probability that the borrower’s default occurs both before the contract’s maturity and the default of the guarantor.

## The standard Gaussian copula and its drawbacks

We now present the Gaussian setting and the related methodological drawbacks for modelling the stochastic dependence among the considered risks.

### The standard Gaussian copula model

In this section, we deal with the evaluation of the theoretical up-front value *V*(*t*) of a defaultable guarantee contract as in (1), using the standard Gaussian copula model first introduced by Li (2001) to financial problems. Without loss of generality, we set $t=0$ hereafter and denote $V(0)=V$.

To assess *V*, we need the joint distribution of the default times of the parties ($\tau _1,\tau _2$) involved in the contract that, according to Sklar’s theorem can be obtained by combining suitable marginal distributions with an appropriate copula function (see, e.g., Meucci 2011, 2011b).

In this analysis we assume constant default intensities for the parties ($\lambda _1,\lambda _2$). Therefore, the default time $\tau _i$ for the $i-th$ party follows a negative exponential marginal distribution. Specifically, the marginal cumulative distribution of the default time $\tau _i$ for the $i-th$ party at time ${\tilde{t}} >0$, namely $F_{i}({\tilde{t}})$, is given by (see, e.g., Lando 1998)

2 $$\begin{aligned} F_{i}({\tilde{t}})=P(\tau _{i} \le \ {\tilde{t}})=1-e^{-\lambda _{i} {\tilde{t}}}\,\,\,\,\,\, \text{ with }\,\,\, i=1,2 \, . \end{aligned}$$

On the other hand, to describe the dependence structure between default times of the parties ($\tau _1,\tau _2$), we use the Gaussian copula. Note that the Gaussian copula has become a market reference for credit risk modelling due to its simplicity and tractability. Indeed, the use of the Gaussian copula is particularly appreciated among practitioners since this copula only needs the linear correlation parameter $\rho$ as input. Then, according to Sklar’s theorem, the joint distribution of the default times $(\tau _{1}$,$\tau _{2})\in {\mathbb {R}}_{+}^{2}$ is obtained by

3 $$\begin{aligned} (\tau _{1},\tau _{2})=(F_{1}^{-1}(u_1),F_{2}^{-1}(u_2)), \end{aligned}$$

where $(u_{1},u_{2})\in \left[ 0,1\right] ^{2}$ are two random vectors generated from a Gaussian copula, and $F_1^{-1}$, $F_2^{-1}$ are the inverse functions of the marginal default times distributions of the parties as in (2). Thus, we can write

4 $$\begin{aligned} (\tau _1,\tau _2)=\biggl (\frac{-\ln (1-u_1)}{\lambda _1},\frac{-\ln (1-u_2)}{\lambda _2}\biggr ) \, . \end{aligned}$$

In the following sections, we report numerical results on the theoretical up-front value *V* as in (1), using the Gaussian model. As we shall see, on the one hand, we can evaluate the up-front value *V* of a defaultable guarantee contract as a function of the correlation parameter $\rho$, which can be easily viewed as the *market* correlation between default intensities. On the other hand, for $\lambda _1 < \lambda _2$, which represents a typical situation, the Gaussian model determines up-front values *V* that are not monotone with respect to the correlation parameter $\rho$, and, therefore, it does not satisfy the so-called *conditions of consistency* described in the Introduction.

### Numerical results for the standard Gaussian model

We provide here numerical results on the theoretical up-front value *V*, as in equation (1), of a defaultable guarantee with maturity $T=3$ years, guaranteed liability $N=100$ euros, and constant recovery rate $R=0.4$. We assume that the dependence structure is described by a Gaussian copula with correlation parameter $\rho$, while the marginal distributions of the default times of the parties are represented by exponential functions with intensities $\lambda _1$ (guarantor), $\lambda _2$ (borrower). We evaluate the up-front value *V* for different levels of the parties’ default intensities ($\lambda _{1}$, $\lambda _{2}$) and for various levels of the copula correlation parameter $\rho$ by using $5 \times 10^{5}$ Monte Carlo simulations. Furthermore, we assume a constant spot rate $r=0.01$. The reference levels of the borrower and of the guarantor intensities have been fixed considering the sovereign and bank CDS market prices as follows: $\lambda _{1}=$ 0.005, 0.01, 0.02, 0.04, 0.06, 0.2; $\lambda _{2}=$ 0.005, 0.01, 0.02, 0.04, 0.06, 0.2. As far as financial market evidence is concerned, these levels correspond to single-name CDS sovereign and bank implied default probabilities observed in the market since 2005 (see Bassan and Mottura 2015). Indeed, empirical evidence shows that credit default swaps spreads can be used as an explanatory variable to assess a sovereign’s creditworthiness (Janus et al. 2013). Even in the banking sector, CDS spreads are significantly related to the credit risk of bank corporations (Kanagaretnam et al. 2016).

**Fig. 1:**
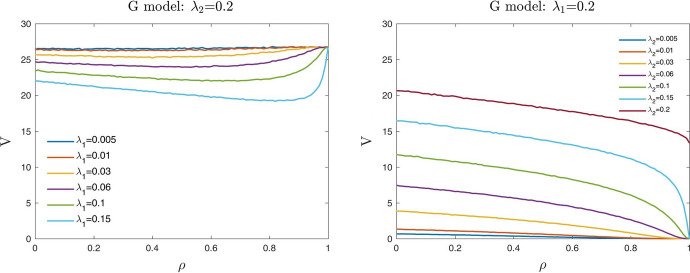
Up-front value *V* (in euros) of a three years guarantee for different values of the default intensity of the guarantor $\lambda _1$ fixed $\lambda _2=0.2$ (left) and of the borrower $\lambda _2$ fixed $\lambda _1=0.2$ (right). G model stands for Gaussian model

In Table 1 and Fig. 1, we report and show the computational results obtained from this empirical setup. As already pointed out, we expect that for a fixed borrower’s creditworthiness $\lambda _2$ and correlation level $\rho$, the value *V* of the guarantee decreases with the weakness of the guarantor $\lambda _1$. Conversely, for a fixed guarantor’s creditworthiness $\lambda _1$ and correlation level $\rho$, *V* increases with the weakness of the borrower $\lambda _2$.

**Table 1:** Up-front value *V* (in euros) of a three years guarantee with a guaranteed liability $N=100$ and a recovery rate $R=0.4$

$\rho$	0.0	0.1	0.2	0.3	0.4	0.5	0.6	0.7	0.8	0.9	1.0
						$$\lambda _2$$					
$\lambda _1$						0.005					
0.005	0.88	0.88	0.85	0.84	0.83	0.81	0.80	0.76	0.70	0.63	0.00
0.01	0.87	0.86	0.85	0.82	0.81	0.77	0.75	0.66	0.58	0.44	0.00
0.02	0.87	0.84	0.80	0.78	0.75	0.71	0.63	0.53	0.42	0.23	0.00
0.04	0.82	0.81	0.76	0.71	0.68	0.60	0.51	0.39	0.26	0.08	0.00
0.06	0.79	0.78	0.73	0.67	0.62	0.53	0.43	0.31	0.17	0.04	0.00
0.2	0.65	0.61	0.53	0.45	0.36	0.28	0.18	0.10	0.03	0.00	0.00
0.01											
0.005	1.77	1.70	1.73	1.67	1.69	1.65	1.62	1.56	1.54	1.49	*1.69*
0.01	1.72	1.72	1.68	1.66	1.65	1.57	1.54	1.44	1.36	1.22	0.00
0.02	1.68	1.66	1.62	1.59	1.52	1.44	1.37	1.23	1.07	0.80	0.00
0.04	1.64	1.59	1.54	1.45	1.36	1.28	1.14	0.96	0.74	0.37	0.00
0.06	1.60	1.54	1.45	1.36	1.26	1.14	0.97	0.79	0.52	0.19	0.00
0.2	1.31	1.20	1.08	0.95	0.78	0.61	0.46	0.26	0.11	0.01	0.00
0.02											
0.005	3.40	3.40	3.37	3.34	3.34	3.31	3.29	3.26	3.25	*3.28*	*3.47*
0.01	3.42	3.37	3.34	3.32	3.22	3.14	3.12	3.06	2.99	2.98	*3.38*
0.02	3.35	3.29	3.26	3.16	3.11	3.00	2.86	2.73	2.58	2.33	0.00
0.04	3.23	3.15	3.09	2.97	2.85	2.69	2.50	2.29	1.95	1.38	0.00
0.06	3.16	3.06	2.94	2.77	2.61	2.44	2.19	1.89	1.48	0.86	0.00
0.2	2.59	2.42	2.21	1.99	1.72	1.42	1.11	0.75	0.38	0.08	0.00
0.04											
0.005	6.67	6.59	6.58	6.54	6.55	6.48	6.48	6.47	*6.55*	*6.59*	*6.66*
0.01	6.56	6.54	6.52	6.42	6.40	6.36	6.28	6.24	*6.28*	*6.45*	*6.68*
0.02	6.51	6.44	6.33	6.29	6.17	6.06	5.96	5.82	5.71	*5.78*	*6.61*
0.04	6.29	6.20	6.06	5.90	5.71	5.56	5.29	5.07	4.76	4.37	0.00
0.06	6.10	5.96	5.81	5.59	5.37	5.18	4.84	4.48	4.00	3.15	0.00
0.2	5.11	4.79	4.42	4.05	3.65	3.21	2.68	2.06	1.31	0.44	0.00
0.06											
0.005	9.70	9.67	9.64	9.63	9.58	9.56	9.54	*9.57*	*9.67*	*9.77*	*9.74*
0.01	9.57	9.53	9.58	9.44	9.44	9.36	9.36	9.30	*9.39*	*9.56*	*9.74*
0.02	9.42	9.36	9.35	9.23	9.14	9.03	8.92	8.82	8.89	*9.11*	*9.82*
0.04	9.17	9.04	8.91	8.71	8.53	8.40	8.16	7.96	7.75	7.70	*9.05*
0.06	8.93	8.77	8.52	8.35	8.04	7.78	7.46	7.16	6.76	6.19	0.00
0.2	7.38	7.01	6.59	6.15	5.65	5.12	4.48	3.67	2.60	1.22	0.00
0.2											
0.005	26.56	26.48	*26.53*	*26.40*	*26.46*	*26.46*	*26.55*	*26.58*	*26.64*	*26.76*	*26.68*
0.01	26.38	26.34	26.22	26.19	*26.34*	*26.35*	*26.36*	*26.54*	*26.62*	*26.68*	*26.61*
0.02	26.00	25.94	25.87	25.74	*25.79*	*25.81*	*25.98*	*26.23*	*26.38*	*26.62*	*26.68*
0.04	25.40	25.22	25.08	24.98	24.87	*24.94*	*24.97*	*25.15*	*25.57*	*26.34*	*26.73*
0.06	24.69	24.41	24.24	24.12	24.08	23.92	*24.03*	*24.18*	*24.67*	*25.62*	*26.70*
0.2	20.75	20.26	19.75	19.33	18.75	18.24	17.66	17.13	16.39	15.49	0.00

Each value represents *V* as a function of the dependence parameter $\rho$, for different values of the default intensity of the guarantor $\lambda _1$ and of the borrower $\lambda _2$, using the Gaussian model. We mark in italics the cases where the value $V$ of the guarantee increases as the parameter $\rho$ increases

Looking at the computational results, we note that when the correlation parameter $\rho$ is equal to 0, the up-front value *V* depends, by definition, only on the marginal risk features of the parties. Accordingly, the values *V* decrease with the default risk of the guarantor $\lambda _1$ when fixing the risk of the borrower $\lambda _2$, and clearly increase in the opposite case. For intermediate levels of correlation, when the default risk of the guarantor is higher than that of the borrower (i.e., $\lambda _1>\lambda _2$), the value *V* always decreases when the correlation parameter $\rho$ increases. On the contrary, if the default risk of the guarantor is lower than that of the borrower (i.e., $\lambda _1<\lambda _2$), the value *V* is not monotonically decreasing w.r.t. the copula’s correlation parameter $\rho$. Indeed, *V* is first decreasing and then, around some correlation levels, starts increasing. This contradictory behaviour can be seen both in Fig. 1 (on the left) and in Table 1. More precisely, in Table 1 we mark in italics the ”non-acceptable” cases where the up-front value *V* of the guarantee increases when the parameter $\rho$ increases. We call “maximum acceptable correlation” ${\bar{\rho }}_{G}$, the maximum level of the copula correlation parameter for which the Gaussian copula (G) model gives realistic results. In other words, the Gaussian model works appropriately for $\rho \le {\bar{\rho }}_{G}$. Furthermore, when the correlation parameter $\rho$ is equal to 1, if the default risk of the guarantor is higher than that of the borrower, then *V* approaches 0. Conversely, if the borrower has a default intensity higher than that of the guarantor (that is the typical situation), then the guarantee’s value *V* reaches its maximum value. This result is exactly the opposite of what one should expect.

### The drawbacks of the standard Gaussian model

Considering the combination between the Gaussian copula and the marginal default time distributions ($F_1,F_2$) with intensities ($\lambda _1,\lambda _2$) as in (2) gives rise to random default times ($\tau _1,\tau _2$) that can be chronologically distant when the copula’s correlation parameter $\rho$ is equal to 1 (Morini 2011). This phenomenon appears in contradiction with the financial meaning of perfect positive correlation. Indeed, for $\rho =1$, the parties should have the same behaviour in terms of default.

This aspect can be shown by using equation (4). We observe that when the copula’s correlation parameter $\rho$ is high ($\rho \rightarrow {1}$) the realisations of ($u_1,u_2$) tend to be equal, i.e., $u_1=u_2=u$. Thus, for $\rho =1$, we have

5 $$\begin{aligned} (\tau _1,\tau _2)=\biggl (\frac{-\ln (1-u)}{\lambda _1},\frac{-\ln (1-u)}{\lambda _2}\biggr ), \end{aligned}$$

hence, the following relation between the default times ($\tau _1,\tau _2$) holds

6 $$\begin{aligned} \tau _{1}=\frac{\lambda _2}{\lambda _1}\tau _2 \, . \end{aligned}$$

As a consequence of (6), the party that defaults first is always the one with the higher default intensity. This implies that if the default intensity of the guarantor is higher than that of the borrower (i.e., $\lambda _1>\lambda _2$), for $\rho =1$ the guarantee’s value *V* approaches to 0, and *V* is always decreasing with respect to the correlation parameter $\rho$. Conversely, if the borrower is the party with the higher default intensity (i.e., $\lambda _1<\lambda _2$), when $\rho =1$, then *V* reaches its maximum value, hence failing the *conditions of consistency*.

**Fig. 2:**
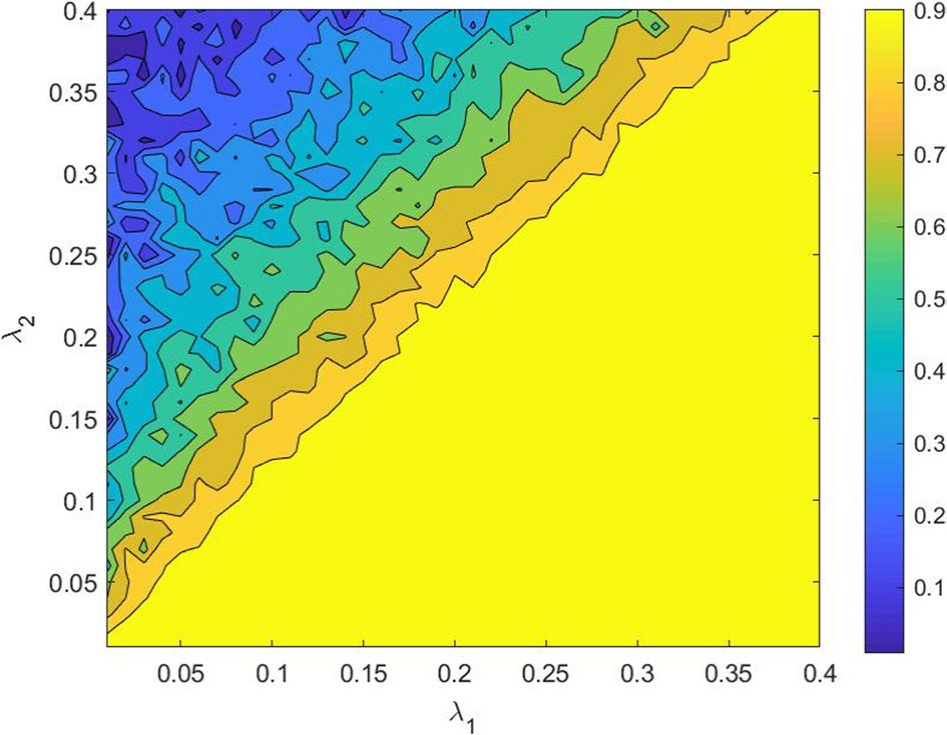
Maximum acceptable correlation ${\bar{\rho }}_G$ as a function of the default intensities of the guarantor $\lambda _1$ and of the borrower $\lambda _2$, using the Gaussian model

In Fig. 2 we show the surface of the “maximum acceptable correlation” ${\bar{\rho }}_{G}$ for the Gaussian model, as a function of different values of the default intensities of the guarantor $\lambda _1$ and of the borrower $\lambda _2$. We observe that the yellow region, where $\lambda _1>\lambda _2$, represents values of ${\bar{\rho }}_{G}$ equal to 1. This means that when the guarantor’s default intensity is higher than that of the borrower, the Gaussian model works appropriately for all $\rho \in [0,1]$. In this case, the up-front value of the guarantee *V* is indeed monotonically decreasing with respect to the correlation parameter $\rho$ and approaches 0 when $\rho =1$ (see Fig. 1 on the right). This result is in line with the *conditions of consistency*. Differently, the darker regions of Fig. 2 identify values of ${\bar{\rho }}_{G}$ less than 1. In particular, the dark blue area represents values of ${\bar{\rho }}_{G}$ that are close to 0. Therefore, in the typical situation where the guarantor is less risky than the borrower (i.e., $\lambda _1<\lambda _2$), the Gaussian model is not able to give realistic results in terms of *V*. In this case, the up-front value of the guarantee *V* is not monotonically decreasing on the copula’s correlation parameter $\rho$, as we should expect, but for $\rho \ge {\bar{\rho }}_{G}$ increases (see Fig. 1 on the left).

## A modified Gaussian model

In this section, we investigate a model that gives rise to consistent values of the guarantee, in the sense of the *conditions of consistency* listed in the Introduction. For this aim, we propose an adjustment of the G model, called Modified Gaussian (MG) model. It is obtained by combining the *standard* Gaussian copula with marginal default time (negative exponential) distributions of the parties where, if $\lambda _{1} < \lambda _{2}$ (i.e., when the Gaussian model does not work consistently), the *market* default intensities ($\lambda _1,\lambda _2$) are appropriately reparametrized in (${\hat{\lambda }}_1,{\hat{\lambda }}_2$) by an “adjustment function” applied to $\lambda _1$.

In the same framework of Sect. 3.1, for the *i*-th part we introduce the *marginal* distribution of the default time $\tau _i$, ${\hat{F}}_{i}({\tilde{t}})$, that is given by

7 $$\begin{aligned} {\hat{F}}_{i}({\tilde{t}})=P(\tau _{i}\le {\tilde{t}})=1-e^{-{\hat{\lambda }}_{i}(\eta _{\lambda },a,c) {\tilde{t}}} \qquad i=1,2 , \end{aligned}$$

where, differently from (2), when $\lambda _{1} < \lambda _{2}$, the “modified” default intensities ${\hat{\lambda }}_{i}(\eta _{\lambda },a,c)$ with $i=1,2$ are

8 $$\begin{aligned} {\hat{\lambda }}_{1}(\eta _{\lambda },a,c)= & {} \lambda _{1}+(\lambda _{2}-\lambda _{1}) \, g(\eta _{\lambda },a,c) \end{aligned}$$

9 $$\begin{aligned} {\hat{\lambda }}_{2}(\eta _{\lambda },a,c)= & {} \lambda _{2} , \end{aligned}$$

always being $\lambda _{1}$ and $\lambda _{2}$ the *market* default intensities. We assume that the function $g(\eta _{\lambda },a,c)$ is an “S” shape curve which for $\eta _{\lambda } \in [0,1]$ ranges from 0 and to 1 in a sigmoidal manner (see the next subsection for an illustrative example of such a function). The constant *a* and *c* determine the shape of *g*, while $\eta _{\lambda }$ is an “adjustment parameter” that is set to appropriately modify the *original* default intensity $\lambda _{1}$ to avoid the drawback of the Gaussian model described in Sect. 3.3.

We point out that the failure of the Gaussian hypothesis can be appreciated only if one takes the data-based calibration of the default intensities. In particular, if $\lambda _1<\lambda _2$ – which represents the standard market condition—and $\rho \rightarrow 1$, then $\tau _1=\frac{\lambda _2}{\lambda _1} \tau _2$, so that $P(\tau _1>\tau _2)=1$. By taking into account the maturity of the contract *T*, one has $P\left[ (\tau _{2}\le T)\cap (\tau _{1}>\tau _{2})\right] =1$. This fact contradicts the mechanism that when $\rho$ grows, the probability that the default of the borrower occurs both before the maturity of the contract and the default of the guarantor $P\left[ (\tau _{2}\le T)\cap (\tau _{1}>\tau _{2})\right]$ should decrease. Since $P\left[ (\tau _{2}\le T)\cap (\tau _{1}>\tau _{2})\right]$ plays a key role in evaluating the theoretical up-front value of the guarantee, this causes the failure of the standard Gaussian model, which substantially does not succeed in modelling the joint default of parties 1 and 2. The introduction of the S-shape adjustment function to the market-based calibrated default intensities allows to model the joint default of the parties; therefore, when $\rho$ is very high, such an adjustment allows to obtain a decrease in the probability that the default of party 2 (borrower) occurs before party 1 (guarantor). In doing so, on the one hand, it is possible to use a benchmark market model—the Gaussian one—and, on the other hand, to satisfy the *conditions of consistency* (1) and (3) (being condition (2) always satisfied by both the standard and the modified models).

Now, as in the Gaussian model, we define the bi-variate variables $(\tau _{1}$,$\tau _{2})\in {\mathbb {R}}_{+}^{2}$ as follows

10 $$\begin{aligned} (\tau _{1},\tau _{2})=({\hat{F}}_{1}^{-1}(u_1),{\hat{F}}_{2}^{-1}(u_2)), \end{aligned}$$

where $(u_{1},u_{2})\in \left[ 0,1\right] ^{2}$ are two random vectors generated by a Gaussian copula with dependence parameter $\rho$, and ${\hat{F}}_{1}^{-1}$, ${\hat{F}}_{2}^{-1}$ are the inverse of the *marginal* distributions of the default times of the parties, having default intensities as in (8) and (9). The joint distribution that characterizes $(\tau _{1}$,$\tau _{2})$ is therefore obtained combining the “modified” *marginal* distributions (7) and a Gaussian copula. We call it the “modified” joint distribution of the default times of the parties, that in the MG model we use for evaluating the up-front value *V* of a defaultable guarantee contract as a function of the dependence parameter $\rho$ and the adjustment parameter $\eta _{\lambda }$, which is used for obtaining consistent values of the guarantee, in the sense of the *conditions of consistency*. The value of $\eta _{\lambda }$ to be used for the specific valuation is a strategic (exogenous) variable in the hand of the calculation agent, which, to be consistent, has to belong to the feasible range of $\eta _{\lambda }$ that defines the set of consistent values of the guarantee for any given $\rho$. Furthermore, for “calibration” purposes, the MG model has to produce values of the guarantee as close as possible to those of the Gaussian model, when the levels of $\rho$ are smaller than the “maximum acceptable correlation” (see Sect. 3.2). Clearly, when $\lambda _{1}<\lambda _{2}$, the “maximum acceptable correlation” can be numerically computed using the Gaussian model, and $0<{\bar{\rho }}_{G}<1$, as shown in Fig. 2.

In the following section, we empirically analyze the surface of the defaultable guarantee contract values *V* as a function of $\rho$ and $\eta _{\lambda }$, highlighting for a fixed value of $\rho$ the corresponding range of admissible values of $\eta _{\lambda }$ for which *V* is consistent. Then, within the feasible range for $\eta _{\lambda }$, we discuss the particular case $\eta _{\lambda }=\rho$, where $\eta _{\lambda }$ becomes an “observable” variable. Clearly, if $\eta _{\lambda }=0$, the results of the MG model collapse on those of the Gaussian model.

### The sigmoid adjustment function

We suppose that

11 $$\begin{aligned} g(\eta _{\lambda },a,c)=\frac{A}{1+e^{-a (\eta _{\lambda }-c)}} + B, \end{aligned}$$

where *A* and *B* are appropriate constants so that $g(0,a,c)=0$ and $g(1,a,c)=1$. Employing some algebraic manipulations, it is straightforward to show that

$$\begin{aligned} A= & {} \frac{\left( 1+e^{-a (1-c)} \right) \left( 1+e^{a c} \right) }{e^{a c} \left( 1-e^{-a } \right) } \\ B= & {} - \frac{ 1+e^{-a (1-c)} }{e^{a c} \left( 1-e^{-a } \right) }\, . \end{aligned}$$

**Fig. 3:**
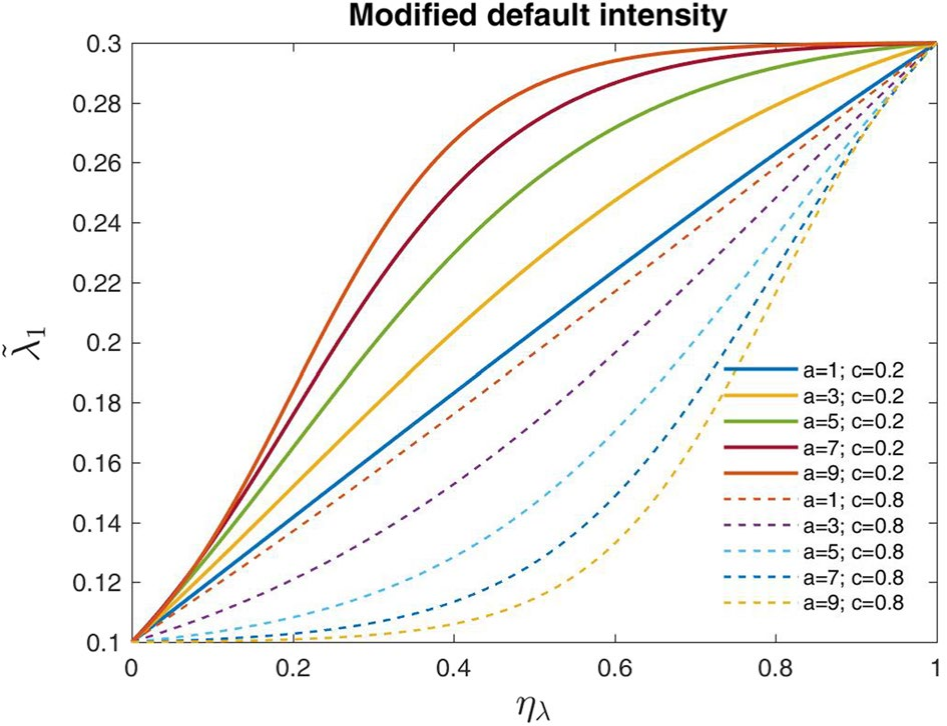
Modified default intensity ${\hat{\lambda }}_{1}(\eta _{\lambda },a,c)$, assuming default intensity of the guarantor $\lambda _1=0.1$ and of the borrower $\lambda _2=0.3$

The constant *c* represents the value of $\eta _{\lambda }$ of the sigmoid’s midpoint, while *a* determines the steepness of the curve. To support intuition in Fig. 3, we show the graph of the adjusted default intensity ${\hat{\lambda }}_{1}(\eta _{\lambda },a,c)$ as a function of the “adjustment parameter” $\eta _{\lambda }$ for several fixed values of the constants *a* and *c*.

It is important to notice that the selection of the sigmoid curve in (11) is not the only possible one. However, this choice fits well with our problem in that the sigmoid function is one of the most versatile and general representations of the adjustment term. Indeed, such a function ranges in a bounded interval, hence including also the situation of no adjustment as a corner case; moreover, it depends on three parameters, whose values allow a large variety of shapes for such a curve.

In the next sections, we will provide some numerical experiments on the Modified Gaussian model. To this aim, we will need to suitably calibrate the parameters of *g* in (11). We now explain the rationale behind the calibration exercise.

We notice that the shape of the curve is remarkably dependent on the selection of parameters *a* and *c*. Reasonably, the selected parameters go in the direction of having a strong similarity between the outcomes of the Gaussian model and those of the Modified Gaussian one when the Gaussian model satisfies the *conditions of consistency*. Under this guide, we take the steepness parameter $a=1$ and the sigmoid’s mid-point parameter *c* equal to the “maximum acceptable correlation” ${\bar{\rho }}_{G}$.

For what concerns the calibration of $\eta _\lambda$, we first detect the feasible variation range of such a parameter which allows the fulfilment of the *conditions of consistency*. Then, two possible solutions could be adopted. The first one is based on expert judgement, considering that the value of $\eta _{\lambda }$ is a strategic (exogenous) variable in the hand of the “valuation” agent. In the European insurance sector, this is a way admitted and widely used in the Solvency II framework, in particular, when adopting the internal model approach (see European Parliament 2009). In the second one, as proposed below, it is $\eta _{\lambda }=\rho$. In this case, the value of the parameter $\rho$ can be calibrated using market data. More precisely, once the model is given, the values of $\rho$ can be implicitly computed by cross-sectional analysis of quoted “multiname” CDS prices at the evaluation date (see Duffie 2004).

Finally, we notice that the three *conditions of consistency* define constraints on the domain of admissibility of the function’s parameters, significantly limiting the “discretion” of the expert’s judgment; even more so in the case $\eta _{\lambda }=\rho$ in which, as already observed, the degrees of freedom of judgment are further reduced.

### Numerical results for the modified Gaussian model

We provide here numerical results of the theoretical up-front value *V* of a defaultable guarantee as a function of $\rho$ and $\eta _{\lambda }$ assuming the default times $(\tau _{1}$,$\tau _{2})$ as in (10). More precisely, we set the maturity $T=3$ years, the guaranteed liability $N=100$ euros, and the recovery rate $R=0.4$. The values *V* are computed for different levels of the *market* default intensities of the parties $\lambda _{1}$ and $\lambda _{2}$ by using $5 \times 10^{5}$ Monte Carlo simulations. Furthermore, we assume *(i)* a constant spot rate $r = 0.01$, and *(ii)* for the sigmoid function *g* the steepness parameter *a* equal to one and the sigmoid’s mid-point parameter *c* equal to the “maximum acceptable correlation” ${\bar{\rho }}_{G}$, in order to obtain values of the MG model as close as possible to those of the Gaussian model (i.e., for calibration purpose).

**Fig. 4:**
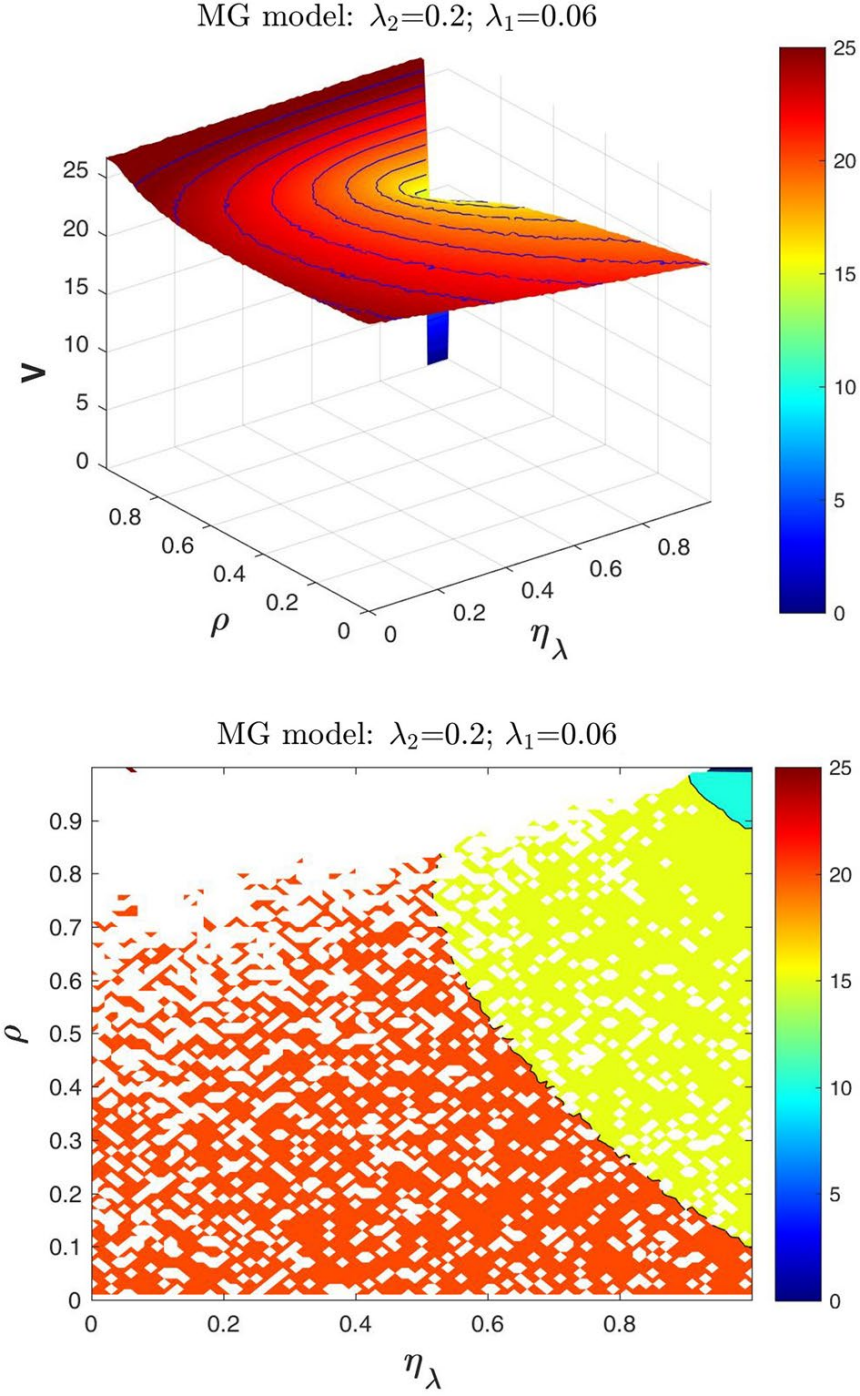
At the top the three-dimensional surface plot of *V* (in euros) as a function of $\rho$ and $\eta _{\lambda }$ of a three years guarantee with $N=100$ and $R=0.4$. At the bottom, the contour plot containing the isolines of *V* only for $\eta _{\lambda }$ values belonging to the feasible range, using the MG model with $\lambda _1=0.06$ and $\lambda _2=0.2$

**Fig. 5:**
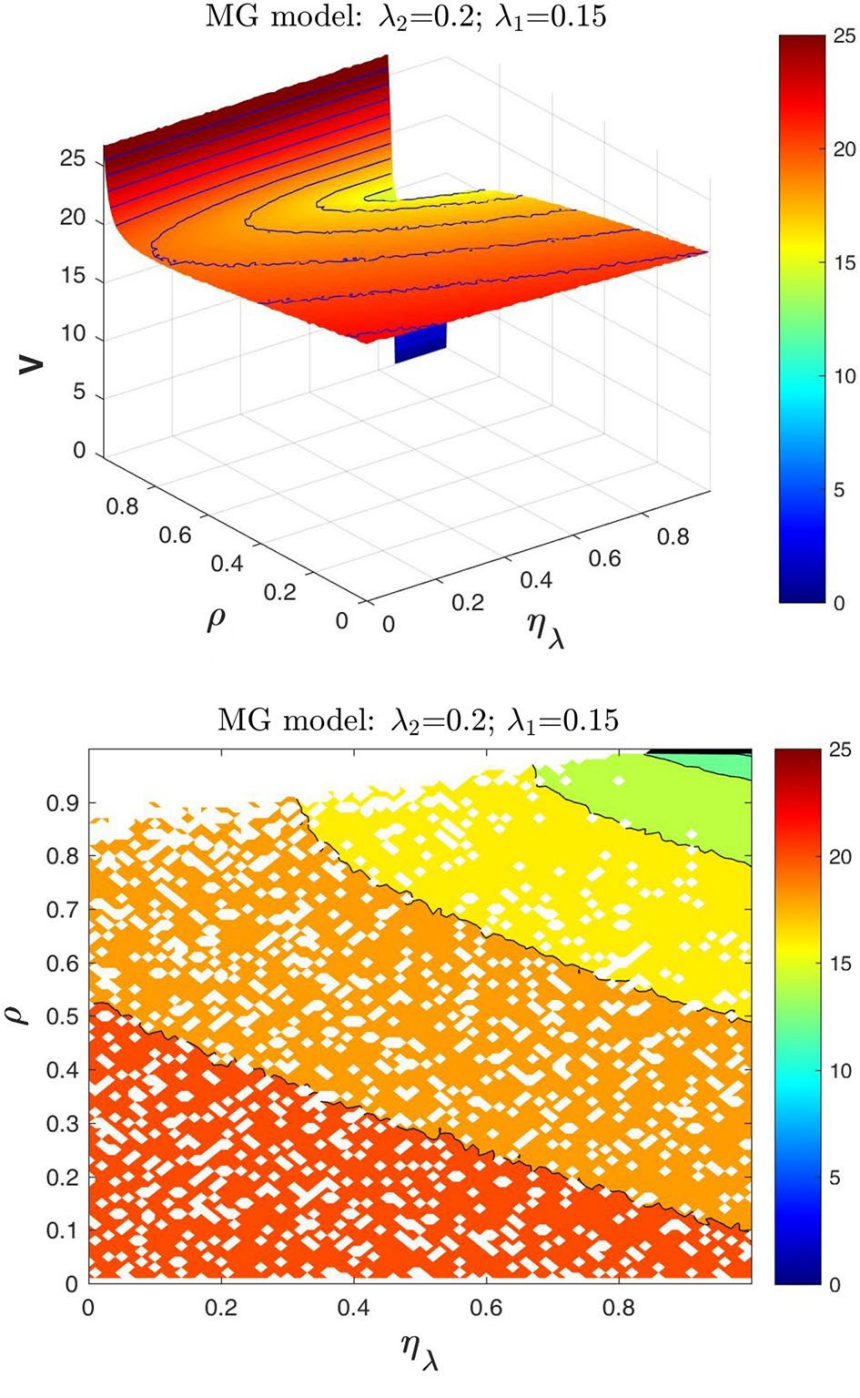
At the top the three-dimensional surface plot of *V* (in euros) as a function of $\rho$ and $\eta _{\lambda }$ of a three years guarantee with $N=100$ and $R=0.4$. At the bottom, the contour plot containing the isolines of *V* only for $\eta _{\lambda }$ values belonging to the feasible range, using the MG model with $\lambda _1=0.15$ and $\lambda _2=0.2$

In Figs. 4 and 5 we show the defaultable guarantee contract value surfaces *V* obtained from the MG model, given the values of the default intensities, by varying the dependence parameter $\rho$ and the adjustment parameter $\eta _{\lambda }$. The values of $\lambda _1$ and $\lambda _2$ are fixed as follows: $\lambda _1=0.06$ and $\lambda _2=0.2$ (Fig. 4); $\lambda _1=0.15$ and $\lambda _2=0.2$ (Fig. 5).

Note that when the adjustment parameter $\eta _{\lambda }$ is equal to 0, the up-front values *V* of the guarantee coincide with those obtained from the standard Gaussian model as a function of $\rho$. The up-front values *V* are decreasing as the parameter $\rho$ increases only for a range of feasible values of the adjustment parameter $\eta _{\lambda }$. Furthermore, the range of feasible values of $\eta _{\lambda }$ decreases when $\rho$ increases. For instance, when $\lambda _1=0.06$ and $\lambda _2=0.2$: if $\rho =0.90$ then the range of feasible values of $\eta _{\lambda }$ is (0.70, 1); if $\rho =0.95$ then the range of feasible values of $\eta _{\lambda }$ is (0.84, 1). We stress here that: (*i*) in general, the value of $\eta _{\lambda }$ to be used for the specific valuation is a strategic (exogenous) variable in the hand of the calculation agent; (*ii*) to be consistent, the value of $\eta _{\lambda }$ has to belong to the $\eta _{\lambda }$ feasible range. In other words, by means of $\eta _{\lambda }$, the *conditions of consistency* reduce the range of possible values of the guarantee.

### A particular case

**Fig. 6:**
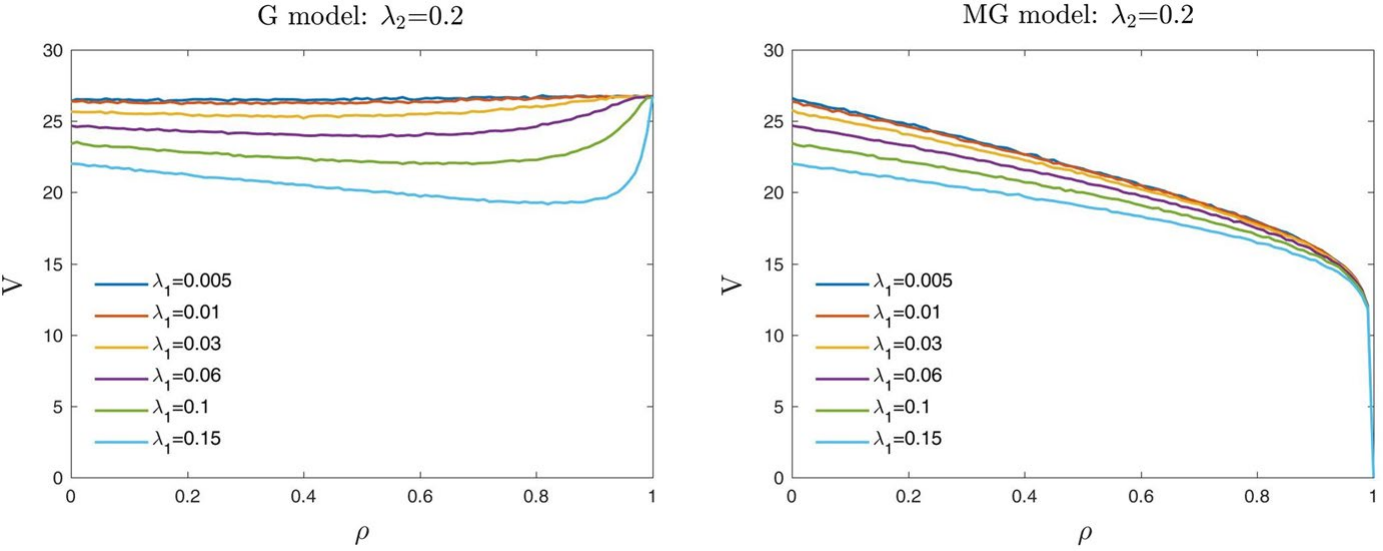
Up-front value *V* (in euros) of a three years guarantee with $N=100$ and $R=0.4$. Each curve represents *V* as a function of $\rho$, for different values of the default intensity of the guarantor $\lambda _1$ when the default intensity of the borrower $\lambda _2=0.2$, using the Gaussian (G) model (left) and the MG model with $\eta _{\lambda } = \rho$ (right)

**Fig. 7:**
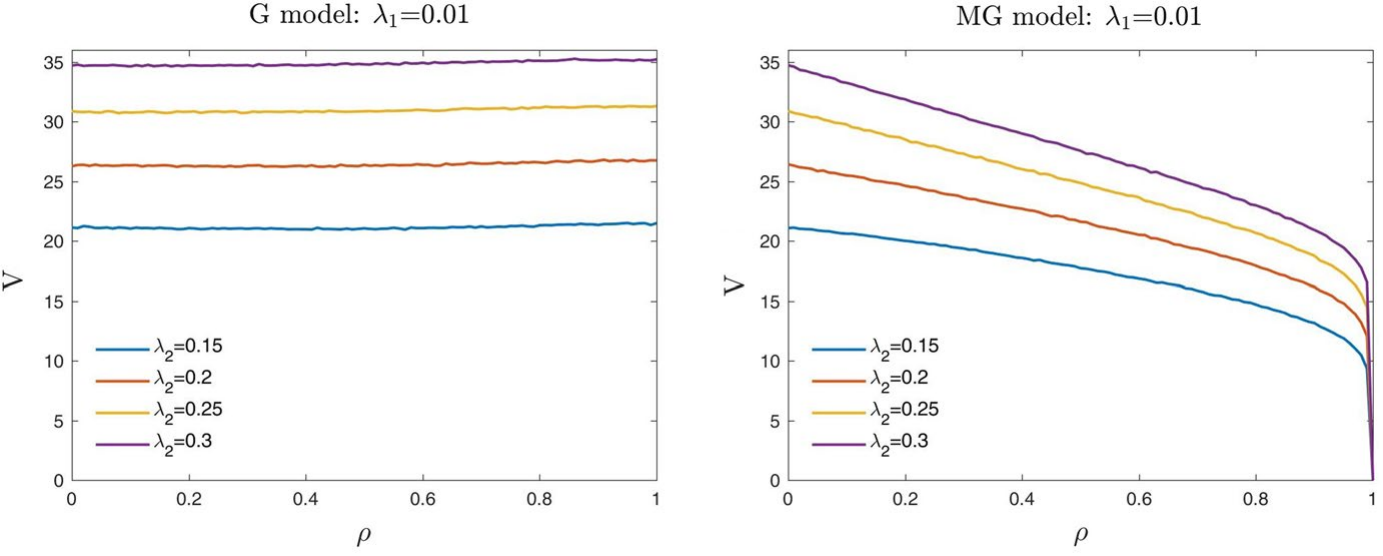
Up-front value *V* (in euros) of a three years guarantee with $N=100$ and $R=0.4$. Each curve represents *V* as a function of $\rho$, for different values of the default intensity of the borrower $\lambda _2$ when the default intensity of the guarantor $\lambda _1=0.01$, using the Gaussian (G) model (left) and the MG model with $\eta _{\lambda } = \rho$ (right)

In this section, given the parameter $\rho$ and within the $\eta _{\lambda }$ feasible range values, we focus on the particular case when $\eta _{\lambda } = \rho$ that in the contour plots at the bottom of Figs. 4 and 5 corresponds to the bisectors. Note that it is a case where $\eta _{\lambda }$ can be interpreted as an “observable” variable. Using the same experimental setup of Sects. 3.2 and 4.2, we report here numerical results for the following reference levels of the borrower and the guarantor default intensities: (i) for $\lambda _{2}=0.2$, $\lambda _{1}=0.005$, 0.01, 0.03, 0.06, 0.1, 0.15 (Fig. 6); (ii) for $\lambda _{1}=0.01$, $\lambda _{2}=0.03$, 0.05, 0.10,0.13, 0.15, 0.2 (Fig. 7).

Figs. 6 and 7 show the computational results obtained for the MG model (on the right) and the standard Gaussian model (on the left).

We notice that (in line with the *conditions of consistency* (1) and (3) described in the Introduction) when the correlation parameter $\rho$ is equal to 0 (i.e., $\eta _{\lambda }=\rho =0$) the up-front value *V* of the guarantee depends only on the marginal risk features of the parties ($\lambda _1,\lambda _2$). Thus, in this case, the values *V* obtained for the MG model are equal to those obtained for the Gaussian model. When the correlation parameter $\rho$ is equal to 1 (i.e., $\eta _{\lambda }=\rho =1$) in the MG model the value *V* always approaches to 0. As requested, for intermediate levels of correlation, using the MG model, the value *V* of the guarantee always decreases when the correlation parameter $\rho$ increases, differently from the Gaussian model when $\lambda _1 < \lambda _2$.

Similar numerical results are reported in Table 2. In line with condition 2 of the *conditions of consistency*, we observe that, given $\rho$ and $\lambda _2$, the values of the guarantee are decreasing with the intensity of default of the guarantor $\lambda _1$, while they are increasing with $\lambda _2$.

**Table 2:** Up-front value *V* (in euros) of a three years guarantee with a guaranteed liability $N=100$ and a recovery rate $R=0.4$

$\eta _{\lambda }=\rho$	0.0	0.1	0.2	0.3	0.4	0.5	0.6	0.7	0.8	0.9	1.0
						$$\lambda _2$$					
$\lambda _1$						0.2					
0.005	26.47	25.74	24.90	23.93	22.94	21.88	20.72	19.56	18.03	16.30	0.00
0.01	26.40	25.47	24.64	23.66	22.74	21.65	20.61	19.41	17.96	16.16	0.00
0.03	25.58	24.91	24.09	23.22	22.25	21.31	20.33	19.10	17.75	16.01	0.00
0.06	24.65	24.02	23.23	22.47	21.62	20.70	19.82	18.69	17.46	15.83	0.00
0.1	23.45	22.75	22.04	21.46	20.79	20.01	19.14	18.23	17.03	15.55	0.00
0.15	22.01	21.52	20.81	20.33	19.65	19.09	18.33	17.55	16.55	15.17	0.00
						$$\lambda _1$$					
$\lambda _2$						0.01					
0.03	5.05	4.96	4.87	4.76	4.66	4.40	4.21	3.92	3.52	2.97	0.00
0.05	8.15	8.04	7.85	7.65	7.39	7.14	6.75	6.29	5.75	4.97	0.00
0.10	15.10	14.85	14.43	13.97	13.51	12.97	12.32	11.57	10.68	9.36	0.00
0.13	18.82	18.41	17.86	17.39	16.63	15.93	15.15	14.25	13.09	11.67	0.00
0.15	21.14	20.63	19.89	19.29	18.46	17.62	16.72	15.79	14.60	13.03	0.00
0.20	26.41	25.53	24.52	23.62	22.70	21.56	20.47	19.28	17.89	16.09	0.00

Each value represents *V* as a function of the dependence parameter $\rho$, for different values of the default intensity of the guarantor and of the borrower, using the MG model with $\eta _{\lambda } = \rho$

For the regulator, it might be useful to identify the “threshold value” of $\rho$, say $\rho ^*$, such that the value of the guarantee issued by the guarantor becomes ineffective for $\rho >\rho ^*$, i.e., the value of the guarantee collapses to 0 (see Figs. 6 and 7). The proposed model and the parameter selection might lead to consider the threshold level $\rho ^*$ as a contractual quantity in the agreement between guarantor and debtor.

#### Some remarks on the meaning of the dependence parameter $\rho$

**Fig. 8:**
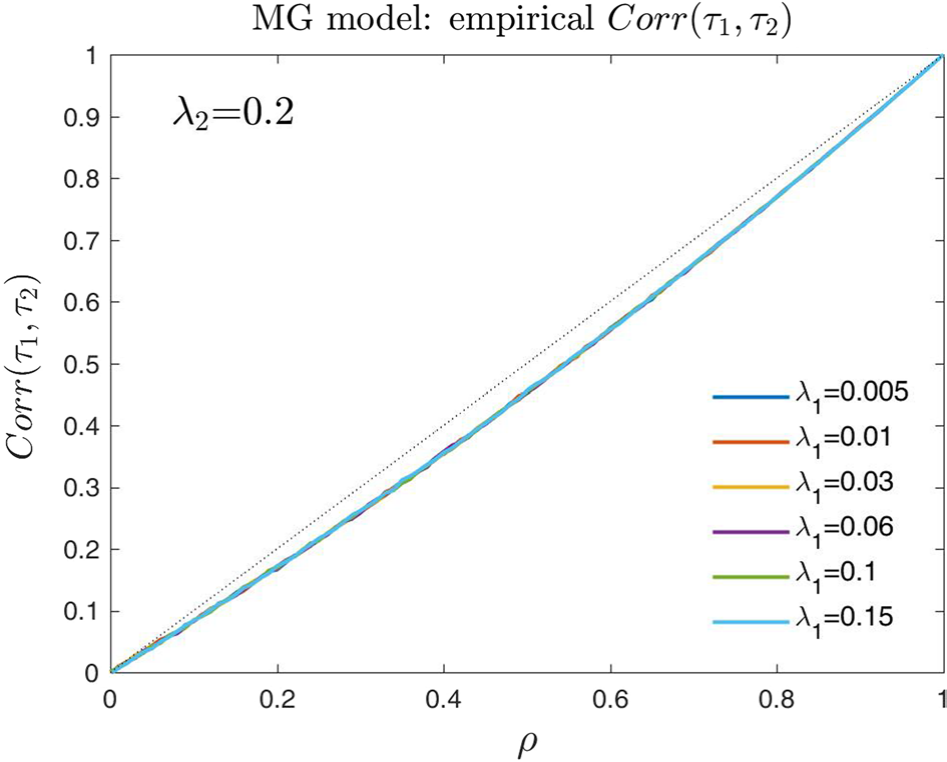
Empirical relation between the dependence parameter $\rho$ and the correlation of the default times $(\tau _1,\tau _2)$, using the MG model with $\eta _{\lambda } = \rho$

We examine here the empirical relation between the parameter $\rho$ and the correlation of the default times $(\tau _1,\tau _2)$ in the particular case where $\eta _{\lambda }=\rho$. More precisely, in Fig. 8 we show the results when $\lambda _{2}=0.2$ and $\lambda _{1}=0.005$, 0.01, 0.03, 0.06, 0.1, 0.15.

We note that the empirical relation between $\rho$ and the correlation of the default times $corr(\tau _1,\tau _2)$ seems to be linear. Furthermore, this empirical relation seems to be invariant to the default intensities of the guarantor $\lambda _1$. Therefore, in the particular case where $\eta _{\lambda }=\rho$ the dependence parameter $\rho$ in the MG model could be still interpreted as a measure of the empirical correlation between the default times for the entire natural domain [0, 1].

## Conclusions

This paper offers a new methodological perspective on Gaussian copulas for financial applications. Specifically, we have addressed the problem of evaluating government guarantees on bank liabilities, focusing on the need for “market-consistent” fees for banks that benefit from such guarantees. We have first presented simulation results obtained by a standard Gaussian copula approach, widely used in the industry to easily interpret its linear correlation parameter. However, we have highlighted some inconsistencies in this model when the default intensity of the guarantor is smaller than that of the borrower. Indeed, in this typical real case, the up-front value of the guarantee is consistent only on a reduced domain of the correlation parameter. Then, to address this issue, we have proposed a generalization of the Gaussian copula (the so-called modified Gaussian copula) that allows for determining realistic results in terms of the guarantees “mark-to-model” value. Indeed, as shown in the computational experiments, such values are consistent on the entire natural domain of the correlation parameter, which also maintains the useful interpretation of the empirical correlation between the default times.

From a financial perspective, future developments might be directed to generalize our approach further by considering a stochastic interest rate intensity for the valuation of the guarantees and stochastic default intensities of the parties for the description of the marginal distributions of the default times. With this aim, new families of copulas might be conceptualized based on the complexity of the financial problem. Furthermore, the Modified Gaussian Copula approach could be adapted to model the stochastic dependence among financial risks in several contexts, including the environment of systemic risk management. In the particular context of the guarantees, one can deal with more complex payoffs and examine theoretical valuation approaches that endogenously allow for the treatment of joint default events.

Finally, we do not aim here at checking whether or not the expected prices produced by the model are in line with the observed prices (as in the back-testing approach). We rather address the problem of pricing based on the information available at the valuation date. The forward-looking evaluation exercise is a future step of our study.
